# Distribution and Influencing Factors of the Sialic Acid Content in the Breast Milk of Preterm Mothers at Different Stages

**DOI:** 10.3389/fnut.2022.753919

**Published:** 2022-03-24

**Authors:** Zekun Chen, Yanmei Chang, Hui Liu, Yanxia You, Yanpin Liu, Xue Yu, Yuqi Dou, Defu Ma, Lijun Chen, Xiaomei Tong, Yan Xing

**Affiliations:** ^1^School of Public Health, Peking University Health Science Center, Beijing, China; ^2^Vanke School of Public Health, Tsinghua University, Beijing, China; ^3^Department of Pediatrics, Peking University Third Hospital, Beijing, China; ^4^National Engineering Center of Dairy for Maternal and Child Health, Beijing Sanyuan Foods Co., Ltd., Beijing, China

**Keywords:** human milk, sialic acid, infant feeding, lactation period, preterm infant

## Abstract

**Background and Aims:**

This study aimed to detect breast milk sialic acid (SA) content and the changing pattern, to understand the various stages of breastfeeding SA secretion, and the influence factors of the human milk SA content.

**Methods:**

We recruited mothers and their infants as our subjects. At days 7, 14, 30, 120, and 365 after delivery, the contents of SA in breast milk were collected and detected through Fluorescence Detector-High Performance Liquid Chromatography. The participants completed the baseline questionnaire at ≤day 7 and were followed up at days 30, 120, and 365.

**Results:**

A total of 95 mothers with 122 infants were included in the analysis, including 22 mothers with 22 term infants, 25 mothers with 35 late preterm infants, 31 mothers with 39 very preterm infants, and 17 mothers with 26 extremely preterm infants. Similar to previous findings, the results of the study showed that, compared with breast milk of term mothers at the same period, breast milk of preterm mothers contained more SA at each time node, and the content of SA in breast milk increased with decreasing gestational weeks. Moreover, maternal age, pre-pregnancy BMI, and delivery mode had significant effects on total SA in breast milk, especially for the preterm infant breast milk. Significant negative associations occurred between SA contents and infant growth status, especially in preterm infants.

**Conclusions:**

We have confirmed the previous observations showing that with the prolongation of lactation time, the content of SA in breast milk gradually decreased, and the content of SA in the breast milk of preterm mothers was higher than that of term mothers. In addition, SA content was associated with maternal age, pre-pregnancy BMI, and delivery mode.

## Introduction

Given the development of modern medical technology and the improvement of obstetric rescue techniques, the morbidity of preterm infants is increasing yearly. The latest data released by the World Health Organization (WHO) in 2019 showed that the global incidence of preterm infants has an average of 10.6% and that the number of preterm infants born in China each year reaches 1.17 million, ranking second in the world (1). Complications of preterm birth are the leading cause of death in children younger than 5 years, accounting for ~35% of deaths among newborn babies. Therefore, prematurity medicine has become a hot spot of medical research.

Breast milk is regarded as the best source of nutrition for full-term infants, and a growing number of studies confirmed the benefits of breastfeeding for preterm infants. At present, most studies on the component of breast milk focused on sugar, fat, protein, minerals, and other nutrients, whereas relatively few studies focused on other bioactive substances in breast milk. Moreover, data on the breast milk component of preterm infant mothers in China are lacking. The amount of each component in the breast milk of term and preterm mothers are different. Gabrielli et al. (2) showed that the oligosaccharide content of preterm milk is higher than that of term milk. With prolonged lactation period, the oligosaccharide content of preterm milk gradually increases, and this finding is consistent with the increased demand of preterm infants for oligosaccharides. Therefore, the bioactive composition of mother's breast milk of preterm infants, such as sialic acid (SA), should be elucidated.

SA, an important functional carbohydrate, is a kind of natural carbohydrate widely existing in the biological system and is a monomer of salivary human milk oligosaccharide (SA human milk oligosaccharides or sialic lactose). SA was first isolated from bovine mandibular salivary gland protein by Blix et al. (3) and named sialic acid. The most common SA is N-acetyl-neuraminic acid, which only exists in the human body and the form of oligosaccharides. The food source of SAs is breast milk, and the SA content is highest in the brain, indicating its importance in human brain cognitive development. Most SAs in breast milk exist in the bound state. About 73.8 and 23.4% of SAs exist in the form of oligosaccharide- and glycoprotein-bound SA, and the remaining 2.8% of SAs exist in the free state (4). However, in formula milk, SAs are bound to protein, accounting for about 70.0% (5). Röhrig et al. (6) reviewed the nutritional effects of sialic acid and confirmed the possibility of applying sialic acid in infant formulas. SAs can promote the physical and mental development of infants. Therefore, it is very important in clarifying the distribution of SAs in breast milk and its effect on offspring's growth and development.

Exogenous SAs are a kind of nutrient widely believed to play a positive role in the growth and development of infants, and their addition into infant formula has become a trend. However, research on SA content and its variation trend in the breast milk of preterm mothers remains lacking. This study dynamically monitors the content, change in total SAs in preterm and term breast milk, and analyzes the relationship between the change in total SAs and the growth, development, and health status of infants, making up the research gap in this field.

## Materials and Methods

### Objective of the Study

This research is part of the Premature Infant Birth Cohort (PIBC) study of Peking University and is sponsored by Beijing Natural Science Foundation (S160004). The PIBC study was a cohort survey carried out in Peking University Third Hospital from December 2017 to June 2019 and investigated the growth and development status of preterm infants at different stages (late, very, and extremely preterm infants). Also, the breast milk of lactating mothers was collected at different follow-up time points to understand the bioactive composition of breast milk of lactating mothers with preterm infants and analyze the growth and development of preterm infants. This study was approved by the Ethics Committee of Peking University Third Hospital (Ethical lot No.260-03) and conformed to the Declaration of Helsinki. Written informed consents were obtained from all participants.

### Inclusion and Exclusion Criteria

The inclusion criteria were as follows: (1) preterm infants with gestational age <37 weeks, daily feeding rate of breast milk of above 50% for 2–3 weeks after birth, then exclusively breastfed for at least 4 months after birth. (2) full-term infants who were exclusively breastfed for at least 4 months after birth (gestational age of 37–42 weeks, singleton, healthy). The exclusion criteria were infants with congenital malformations, genetic diseases, no realistic hope of survival, or infants requiring surgery.

### Data Collection and Variable Definitions

The participants completed the baseline questionnaire at 7 days or less and were followed up at 30, 120, and 365 days. In the baseline study, the basic characteristics of mothers, such as ages, parity, delivery mode, education level, and income were investigated. Clinical examinations, such as height and weight were performed, and the anthropometric data of infants (i.e., weight, length, and head circumference) were measured. Anthropometric measurement was carried out in accordance with the standard measurement method by the pediatrician. Staffs were trained uniformly, and the procedure was standardized. Infant weight and length were measured on an electronic scale with accuracy of 5 g and 1 cm, and the infant head circumference was measured using tape. Maternal age, height, and weight were self-reported at different follow-up time points. At each subsequent visit, clinical examinations were performed, and anthropometric measurements of infants were obtained.

### Sample Collection and Analysis

Breast milk samples of mothers at different postpartum lactation stages were collected dynamically. Breast milk was collected at 7, 14, 30, 120, and 365 days after birth in accordance with a unified process. After the breast was sterilized, one side of the breast was emptied in the morning (9:00 a.m.) and mixed. About 5–10 ml of milk was collected and stored in a refrigerator maintained at −80°C for examination. In this study, the Fluorescence Detector–High-Performance Liquid Chromatography was used to determine the total SA content in breast milk, which was the sum of free SAs and oligosaccharide-, glycoprotein-, and casein-bound SAs.

### Statistical Analysis

Subjects were coded uniformly. The EpiData software (EpiData version 3.1, The EpiData Association Odense, Denmark) was used for data entry in parallel pairs. The SPSS26.0 (SPSS Inc., Chicago, IL, USA) and R (version 3.4.4) were used for statistical analysis. Continuous data were expressed as means ± standard deviation (SD), and proportion was used to describe the categorical data. One-way analysis of variance was used to verify differences among groups. The chi-square test was conducted to compare differences in categorical variables among groups. The Pearson correlation test was used to validate associations between SA concentrations and infant characteristics, including infants' weight and height. *P* < 0.05 (two-tailed) was considered significant in all analyses.

## Results

**Figure 1:**
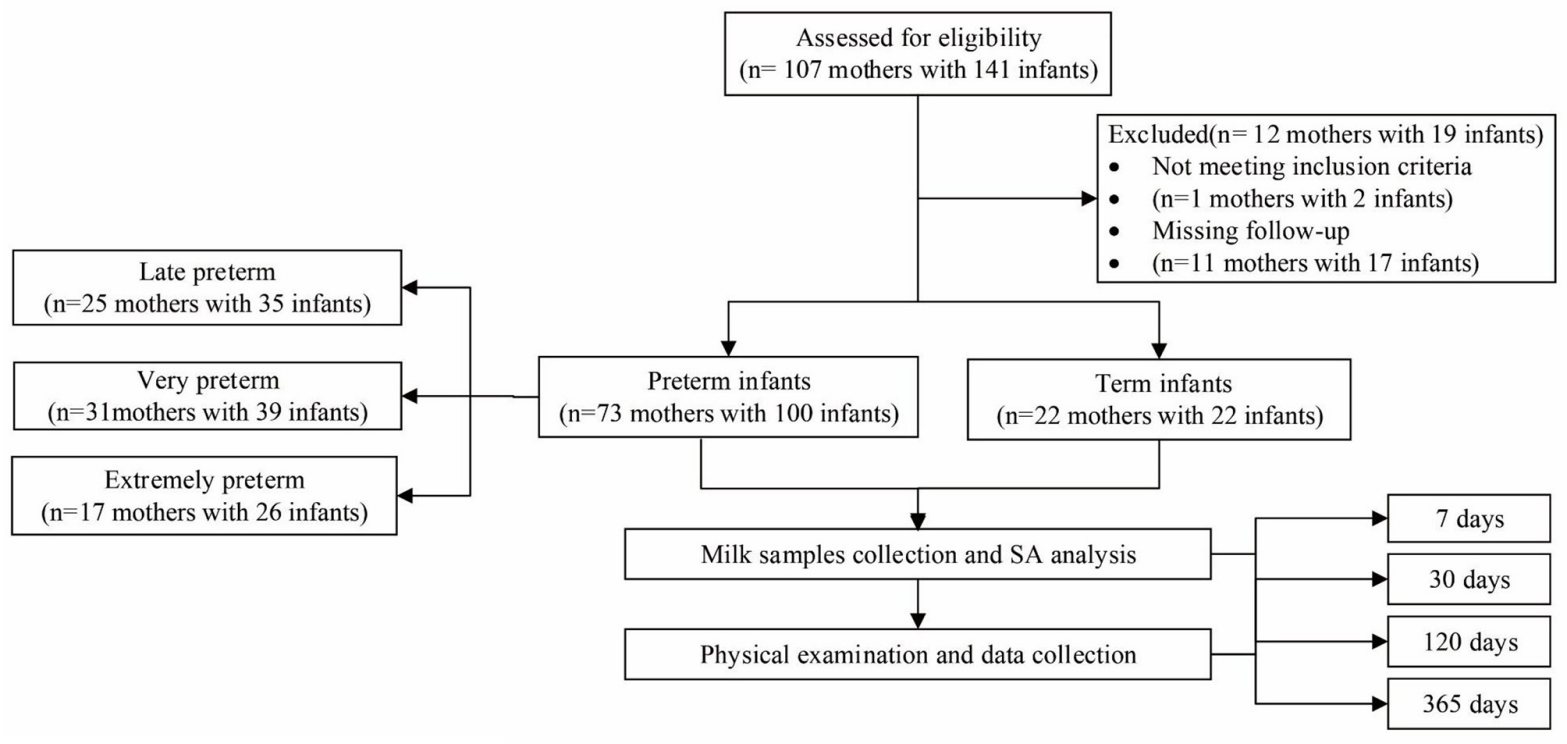
Flow chart of inclusion.

A total of 107 mothers with 141 infants were enrolled in this study, and 95 mothers with 122 infants with sufficient data were included in the analysis, including 22 mothers with 22 term infants, 25 mothers with 35 late preterm infants, 31 mothers with 39 very preterm infants, and 17 mothers with 26 extremely preterm infants. Figure 1 presents the study flow. Two mothers were excluded because they did not meet the inclusion criteria, and 10 mothers were dropped out of the study because they were lost to follow-up. No serious adverse event was reported during the study period.

Table 1 summarizes the baseline anthropometric and demographic data of mothers and infants. The maternal baseline characteristics of each group were comparable. Assisted conception was found frequently in preterm mothers. Cesarean sections were common in late (82.86%) and very (74.36%) preterm infants. Significant differences were observed between preterm and term infants on all baseline parameters except gender distribution. Moreover, multiple births were common in preterm infants (60.00, 41.03, and 69.23% for late, very, and extremely preterm infants, respectively).

**Table 1:** The basic characteristics of mothers and infants (mean ± SD for continuous data and proportion for categorical data).

**Variables**	**Term**	**Late preterm**	**Very preterm**	**Extremely preterm**	***P***
Mother	22	25	31	17	
Maternal age (year)	32.04 ± 3.64	33.06 ± 3.67	33.25 ± 5.17	33.41 ± 3.32	0.697
Pre-pregnancy BMI (kg/m^2^)	21.62 ± 3.73	22.63 ± 3.02	22.25 ± 3.54	23.49 ± 3.38	0.341
Primiparous (%)	63.64%	70.59%	73.68%	85.00%	0.469
Mother's education (%)					0.367
College/University	63.64%	60.00%	50.00%	59.09%	
Postgraduate and above	36.36%	28.57%	26.47%	22.73%	
Assisted conception (%)	18.18%	20.59%	42.11%	70.00%	**0.002**
Cesarean section (%)	45.45%	82.86%	74.36%	7.69%	**<0.001**
Infant	22	35	39	26	
Boys (%)	50.00%	48.57%	53.85%	53.85%	0.996
Multiple births (%)	0.00%	60.00%	41.03%	69.23%	**<0.001**
Birth weight (kg)	3.40 ± 0.35	2.00 ± 0.41	1.26 ± 0.29	0.92 ± 0.09	**<0.001**
Birth length (cm)	49.68 ± 2.03	43.21 ± 3.32	37.84 ± 3.98	34.72 ± 2.16	**<0.001**
Birth head circumference (cm)	34.23 ± 1.48	31.06 ± 1.90	27.49 ± 2.41	24.28 ± 1.27	**<0.001**

*Bold p-values are statistically significant*.

**Figure 2:**
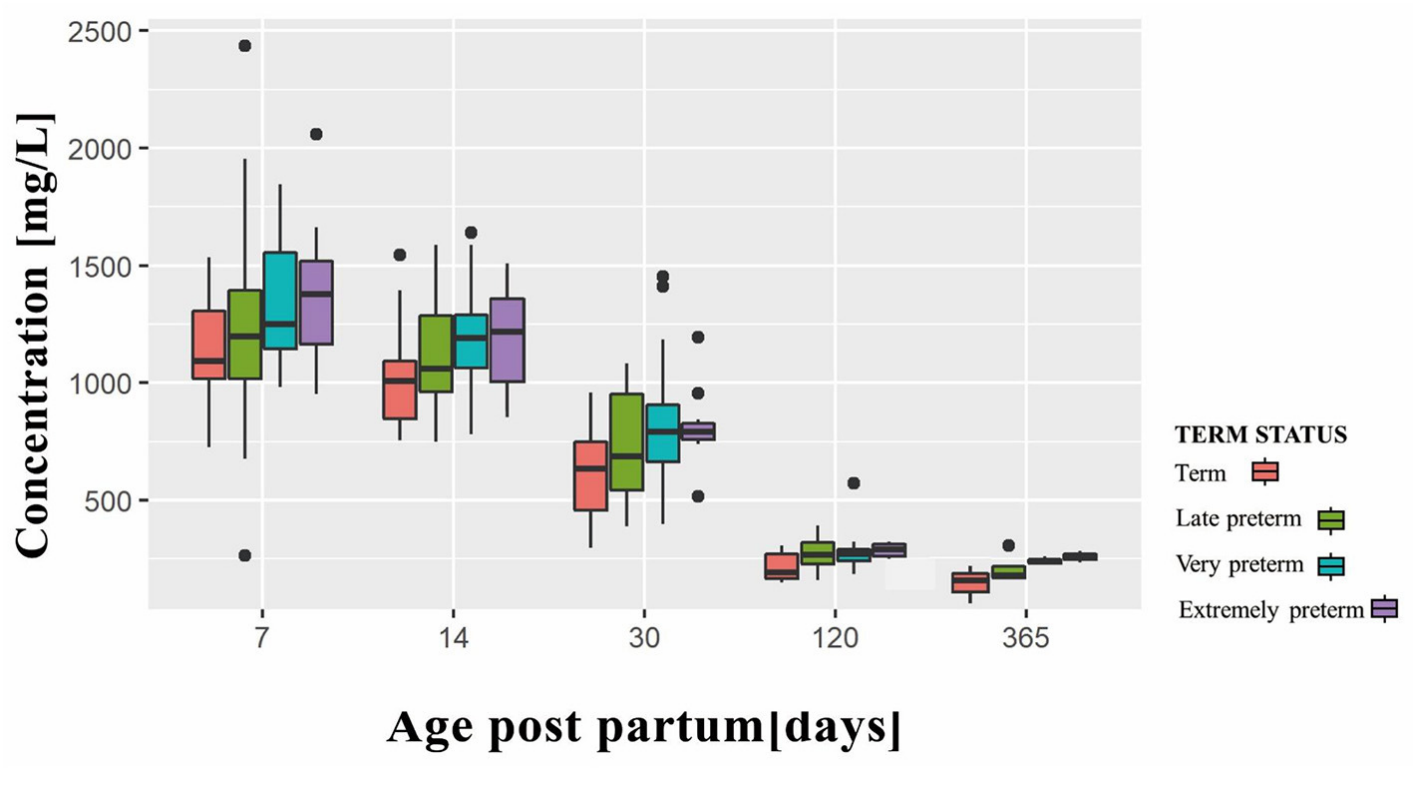
Variation trend of sialic acid over time.

Figure 2 shows the changing trend in the SA content in different preterm and term infant groups over time. As shown in Figure 2, significant differences were observed in the SA content in breast milk at 7, 14, 30, 120, and 365 days postpartum (*P* < 0.05). The highest concentration of SA in the term infant group was observed 7 days after birth, which then decreased dramatically during lactation. Preterm infant groups presented a similar trend. Moreover, compared with term infant breast milk at the same period, preterm breast milk contained more SA at each time node. In addition, the content of SA in breast milk increased with decreasing gestational weeks, and the difference was statistically significant.

Table 2 displays the distribution of SA components in breast milk under different variables. In accordance with the gestational age of infants, infants were divided into term and preterm infants. Preterm infant group results showed significant differences in SA levels among mothers of different ages at days 14 and 30, and the highest SA secretion was observed in mothers over the age of 35, which were 1,227.27 ± 278.52 mg/L and 879.41 ± 253.74 mg/L, respectively. The BMI stratified analysis showed that mothers with BMI ≥ 24.0 kg/m^2^ had higher SA content in their breast milk than mothers with BMI < 24.0 kg/m^2^, and significant differences were observed in the SA secretion levels between the two groups on days 14, 120, and 365 (*P* < 0.05). Moreover, breast milk from mothers who delivered naturally had higher SA levels at day 7 than that from mothers who delivered by cesarean section (*P* = 0.004). The stratified analysis showed considerable influencing factors in the total group were mainly reflected in preterm infants. The total group showed that the SA contents in the breast milk of mothers with BMI ≥ 24.0 kg/m^2^ at days 14 (*P* = 0.014) and 120 (*P* < 0.001) were higher than that in mothers with BMI < 24.0 kg/m^2^. The SA content in the breast milk of mothers who delivered naturally 7 days after birth was higher than that of mothers who delivered by cesarean section (*P* = 0.017).

**Table 2:** Distribution of sialic acid contents in breast milk under different variables (mg/L, Mean ± SD).

**Sialic acid**	**Day 7**	***P***	**Day 14**	***P***	**Day 30**	***P***	**Day 120**	***P***	**Day 365**	***P***
**Preterm infants**										
**Maternal age, year**		0.091		**0.014**		**0.011**		0.274		0.637
≤ 30	1,338.06 ± 363.05		1,117.21 ± 146.56		808.09 ± 193.76		247.11 ± 74.48		189.50 ± 37.23	
31–35	1,198.59 ± 308.99		1,070.49 ± 124.72		715.89 ± 151.36		291.25 ± 86.46		246.09 ± 49.88	
>35	1,390.32 ± 309.93		1,227.27 ± 278.52		879.41 ± 253.74		280.31 ± 42.43		234.99 ± 56.35	
**Pre-pregnancy BMI, kg/m** ^**2**^		0.205		**0.027**		0.185		**<0.001**		**0.002**
BMI <24.0	1,266.42 ± 376.14		1,098.99 ± 189.86		773.90 ± 191.82		254.58 ± 56.57		187.18 ± 26.52	
BMI ≥ 24.0	1,368.48 ± 204.40		1,200.99 ± 199.31		838.05 ± 232.81		322.73 ± 76.08		268.53 ± 28.84	
**Parity**		0.838		0.700		0.161		0.274		NA
Primiparity	1,287.09 ± 366.28		1,138.60 ± 171.90		808.44 ± 209.95		288.54 ± 79.49		NA	
Pluriparity	1,306.99 ± 304.40		1,118.01 ± 276.89		730.51 ± 212.26		264.92 ± 42.82		NA	
**Delivery mode**		**0.004**		0.862		0.722		0.431		0.116
Natural delivery	1,420.49 ± 343.14		1,138.90 ± 166.30		800.77 ± 198.37		287.98 ± 80.53		266.47 ± 23.41	
Cesarean section	1,210.71 ± 308.53		1,131.05 ± 218.87		785.10 ± 210.91		271.49 ± 64.62		215.67 ± 53.51	
**Mother's education**		0.733		0.253		0.699		0.087		**0.018**
College/University and below	1,288.97 ± 372.55		1,119.77 ± 197.82		786.79 ± 190.38		263.16 ± 61.90		201.97 ± 40.27	
Postgraduate and above	1,318.12 ± 197.46		1,178.40 ± 197.07		806.06 ± 246.00		298.31 ± 79.08		270.02 ± 31.98	
**Income**		0.736		0.831		0.816		0.955		0.557
<8,000	1,228.90 ± 310.72		1,117.30 ± 244.14		757.19 ± 315.37		274.78 ± 88.85		216.05 ± 63.74	
≥8,000	1,272.45 ± 404.92		1,132.97 ± 219.58		775.10 ± 216.72		276.41 ± 71.05		240.98 ± 49.42	
**Infant sex**		0.655		0.113		0.268		0.339		0.977
Boy	1,278.52 ± 282.22		1,098.00 ± 194.31		766.01 ± 211.60		286.38 ± 81.37		235.58 ± 38.41	
Girl	1,311.36 ± 381.43		1,167.91 ± 197.80		814.21 ± 197.97		266.39 ± 52.93		236.61 ± 70.18	
**Term infants**										
**Maternal age, year**		0.463		0.491		0.890		0.696		NA
≤ 30	1,132.84 ± 268.24		1,016.85 ± 228.32		607.46 ± 238.10		233.81 ± 59.99		NA	
31–35	1,018.51 ± 82.80		907.75 ± 103.79		572.65 ± 170.49		207.82 ± 56.32		NA	
>35	1,145.87 ± 190.40		1,048.41 ± 301.89		554.25 ± 128.86		213.76 ± 59.69		NA	
**Pre-pregnancy BMI, kg/m** ^**2**^		**0.033**		0.700		0.092		0.503		NA
BMI <24.0	1,042.39 ± 163.29		973.29 ± 206.83		543.28 ± 178.76		216.54 ± 56.05		NA	
BMI ≥ 24.0	1,257.15 ± 228.52		1,017.64 ± 248.79		708.69 ± 185.17		241.44 ± 66.24		NA	
**Parity**		0.669		0.891		0.733		0.249		NA
Primiparity	1,112.25 ± 203.28		979.65 ± 185.61		573.58 ± 213.35		208.13 ± 50.20		NA	
Pluriparity	1,071.82 ± 204.50		994.07 ± 268.04		605.16 ± 150.75		240.44 ± 64.08		NA	
**Delivery mode**		0.796		**0.048**		0.215		0.383		NA
Natural delivery	1,084.06 ± 228.37		1,081.26 ± 258.71		638.41 ± 210.53		232.70 ± 62.11		NA	
Cesarean section	1,108.10 ± 177.29		898.29 ± 113.99		530.86 ± 159.99		208.68 ± 51.04		NA	
**Mother's education**		0.987		0.620		0.363		0.526		NA
College/University and below	1,096.69 ± 228.26		963.52 ± 203.81		613.86 ± 213.08		214.47 ± 57.50		NA	
Postgraduate and above	1,095.17 ± 161.24		1,014.45 ± 234.07		530.35 ± 135.75		233.13 ± 57.52		NA	
**Income**		0.219		0.466		0.801		0.630		NA
<8,000	1,339.85 ± 127.24		828.98 ± 183.43		535.99 ± 157.13		193.13 ± 28.53		NA	
≥8,000	1,083.25 ± 196.16		993.63 ± 214.92		587.19 ± 195.03		222.31 ± 57.82		NA	
**Infant sex**		0.255		0.923		0.887		0.565		NA
Boy	1,147.93 ± 170.73		980.28 ± 176.79		578.36 ± 200.13		212.71 ± 50.37		NA	
Girl	1,044.23 ± 220.86		990.17 ± 257.20		590.91 ± 190.28		228.67 ± 64.09		NA	
**Total**										
**Maternal age, year**		0.069		**0.009**		**0.033**		0.328		0.401
≤ 30	1,298.97 ± 353.59		1,098.22 ± 166.07		767.05 ± 216.64		241.20 ± 66.82		197.43 ± 11.21	
31–35	1,167.07 ± 290.17		1,036.98 ± 136.66		690.82 ± 162.19		272.71 ± 87.23		266.06 ± 66.21	
>35	1,339.39 ± 302.93		1,196.43 ± 285.42		829.39 ± 265.31		268.21 ± 51.55		248.59 ± 57.49	
**Pre-pregnancy BMI, kg/m** ^**2**^		0.104		**0.014**		0.114		**<0.001**		**<0.001**
BMI <24.0	1,222.20 ± 355.27		1,072.72 ± 198.69		730.66 ± 208.99		242.44 ± 58.60		190.81 ± 24.37	
BMI ≥ 24.0	1,349.93 ± 208.71		1,173.21 ± 213.80		816.49 ± 228.01		310.54 ± 78.89		285.01 ± 42.52	
**Parity**		0.734		0.649		0.158		0.573		0.806
Primiparity	1,258.35 ± 349.81		1,109.26 ± 183.83		768.27 ± 227.26		267.97 ± 80.77		254.25 ± 34.03	
Pluriparity	1,231.73 ± 294.12		1,087.02 ± 275.21		696.76 ± 202.75		257.47 ± 50.03		245.37 ± 73.15	
**Delivery mode**		**0.017**		0.401		0.586		0.427		0.806
Natural delivery	1,345.73 ± 348.80		1,126.55 ± 187.66		766.94 ± 209.54		270.83 ± 78.61		254.25 ± 34.03	
Cesarean section	1,193.89 ± 292.41		1,090.92 ± 222.30		744.10 ± 223.28		256.61 ± 66.75		245.37 ± 73.15	
**Mother's education**		0.973		0.445		0.716		**0.048**		0.092
College/University and below	1,259.00 ± 359.65		1,095.90 ± 205.25		758.69 ± 203.22		248.55 ± 64.00		218.80 ± 54.74	
Postgraduate and above	1,256.62 ± 211.27		1,131.56 ± 217.31		741.73 ± 252.56		283.83 ± 78.80		274.48 ± 53.75	
**Income**		0.778		0.883		0.685		0.694		0.421
<8,000	1,237.43 ± 299.08		1,096.71 ± 246.89		742.44 ± 309.22		266.61 ± 87.66		216.05 ± 63.74	
≥8,000	1,207.09 ± 356.85		1,086.53 ± 225.95		713.54 ± 226.35		256.42 ± 70.90		254.73 ± 59.78	
**Infant sex**		0.860		0.134		0.271		0.426		0.826
Boy	1,252.40 ± 267.45		1,073.97 ± 195.06		729.92 ± 220.55		268.94 ± 81.04		245.21 ± 43.33	
Girl	1,263.66 ± 371.04		1,136.54 ± 217.60		775.71 ± 212.78		254.68 ± 58.22		252.95 ± 78.58	

*NA: not available, too few data elements in one group. Bold p-values are statistically significant*.

Table 3 presents the Pearson correlation of SA content and infant growth status. In this study, SA was not associated with term infant growth status and inversely associated with weight and body length development in preterm infants. In preterm infants, the SA content was negatively associated with infant weight and body length. The correlation decreased with prolonged lactation time, and data were only significant at days 7 and 30 but not significant at days 120 and 365 (*P* < 0.05).

**Table 3:** Pearson correlation of sialic acids content and infant growth status.

**Preterm infants**	**Sialic acid**	***P***	**Term infants**	**Sialic acid**	***P***	**Total**	**Sialic acid**	***P***
Day 7 WG	−0.300	**0.005**	Day 7 WG	0.057	0.811	Day 7 WG	−0.421	**<0.001**
Day 7 LG	−0.249	**0.030**	Day 7 LG	−0.065	0.784	Day 7 LG	−0.382	**<0.001**
Day 30 WG	−0.229	**0.030**	Day 30 WG	−0.066	0.781	Day 30 WG	−0.373	**0.003**
Day 30 LG	−0.250	**0.041**	Day 30 LG	−0.090	0.740	Day 30 LG	−0.412	**0.002**
Day 120 WG	−0.046	0.753	Day 120 WG	−0.233	0.351	Day 120 WG	−0.273	**0.032**
Day 120 LG	−0.097	0.553	Day 120 LG	−0.164	0.545	Day 120 LG	−0.337	**0.011**
Day 365 WG	−0.341	0.334	Day 365 WG	0.500	0.667	Day 365 WG	0.187	0.187
Day 365 LG	−0.398	0.255	Day 365 LG	0.246	0.294	Day 365 LG	0.113	0.713

*WG, Weight gain; LG, Length gain. Bold p-values are statistically significant*.

Table 4 presents the effects of different levels of SA on the growth and development of infants. In this study, the mean SA content of breast milk in different gestational weeks was considered as the boundary, and breast milk SA was divided into low- and high-SA groups. In the preterm infant group, infants in the low-SA group had higher body length and weight at days 7 and 30 than those in the high-SA group (*P* < 0.05). However, these differences were not significant at days 120 and 365, which suggested that high levels of SA in breast milk might useful for the catch-up growth of preterm infants.

**Table 4:** Effects of different levels of sialic acids on the growth and development of infants.

**Sialic acid**	**Length (cm)**	***P***	**Weight (kg)**	***P***
**Preterm infants**				
**7 days**		**0.014**		**0.045**
Low	40.31 ± 4.69		1.55 ± 0.59	
High	38.38 ± 5.02		1.30 ± 0.43	
**30 days**		**0.046**		**0.016**
Low	49.79 ± 5.41		3.06 ± 0.87	
High	48.29 ± 4.47		2.76 ± 0.75	
**120 days**		0.479		0.341
Low	59.33 ± 4.59		6.35 ± 1.34	
High	57.86 ± 5.18		5.90 ± 1.20	
**Term infants**				
**7 days**		0.762		0.917
Low	49.62 ± 2.36		3.43 ± 0.40	
High	50.00 ± 0.82		3.41 ± 0.11	
**30 days**		0.817		0.801
Low	57.27 ± 2.60		5.13 ± 0.76	
High	56.87 ± 3.01		5.03 ± 0.32	
**120 days**		0.196		0.313
Low	66.52 ± 3.60		7.71 ± 1.06	
High	63.00 ± 6.96		7.17 ± 0.98	
**Total**				
**7 days**		**0.012**		**0.004**
Low	43.62 ± 6.02		2.18 ± 1.04	
high	39.93 ± 6.16		1.54 ± 0.80	
**30 days**		**0.023**		**<0.001**
Low	53.53 ± 5.64		3.89 ± 1.31	
High	49.29 ± 4.87		2.95 ± 0.96	
**120 days**		0.093		0.063
Low	62.76 ± 5.60		7.02 ± 1.40	
High	59.47 ± 6.07		6.26 ± 1.26	

*Bold p-values are statistically significant*.

## Discussion

Few studies clarified the SA level in the breast milk of preterm mothers at different stages. In this cohort study, we compared the differences in breast milk SA levels among different gestational age preterm and term mothers at different stages after delivery for the first time. The SA concentrations in the breast milk of lactating mothers at 7, 14, 30, 120, and 365 days postpartum were studied. We found that the content of SAs in the breast milk of preterm and term mothers decreased with prolonged lactation time. In addition, preterm breast milk contained more SA than term breast milk at each time node, and the content of SA in breast milk significantly increased with decreasing gestational weeks.

The results of this study showed that among the breast milk of neonatal mothers at 7, 14, 30, 120, and 365 days after delivery, the SA content in breast milk on day 7 was the highest. With the extension of the lactation period, the SA content in breast milk gradually decreased, and this finding was similar to those of previous studies. Wang et al. (4) reported that the SA concentration in breast milk shows a dynamic trend throughout the lactation period. With the extension of lactation, the breast milk of SA content gradually decreases, which may be the result of the long-term evolution of the human lactation mechanism. The synthesis of SAs is carried out in the liver (7, 8). The study of Duncan et al. (9) showed that in newborn mice, the ability to synthesize SAs is weak, and exogenous SAs should be absorbed to meet the needs of normal growth and development. With the growth of age, the liver function of the infant gradually improves, and its ability to synthesize SAs is enhanced with age. Thus, the demand for exogenous SAs may be reduced. This finding suggests that the change in the SA level of lactating mothers is the self-adjustment by the growth and development of infants, and the different utilization rates of SA in infants at different periods of lactation may lead to different SA levels in breast milk. The dynamic changes in SA secretion from mother's milk may be determined by different lactation requirements.

In this study, we found that the SA content in preterm breast milk was significantly higher than that in term breast milk at each time node and that the content of SAs in breast milk increased with decreasing gestational weeks. Exogenous SAs can increase the content of SAs in brain tissue, which is conducive to the development of the brain and the improvement of cognitive ability (10, 11). For newborns, milk is the only source of exogenous SAs (12, 13). Early breast milk has rich SA content, which is conducive to brain development (14). Preterm infants are born with immature brain development and often have to be hospitalized for a long time due to various reasons. At present, the hospitalization mechanism of newborns in many countries cannot achieve mother–infant co-living. Thus, these preterm infants cannot get colostrum or transition milk from their mothers and lose the opportunity to get high concentration of exogenous SAs. This phenomenon may result in a loss of brain development in preterm infants.

This study suggested that maternal age, pre-pregnancy BMI, and delivery mode had significant effects on total SA in breast milk. Old age and mothers with BMI ≥ 24.0 kg/m^2^ had high levels of SAs in their breast milk, that might be because obesity and overweight are significantly associated with increased risk of preterm birth and with increased SA content in serum (15) and other biological fluids. Moreover, mothers who delivered naturally had higher total SA in breast milk at each time point than mothers who delivered by cesarean section, confirming the superiority of natural labor.

Studies investigating how SAs are related to infant growth are sparse. In the present study, a significant difference in infant weight and body length in the first month after birth was observed between high- and low-SA groups of preterm infants. SA was suggested to play an important role in the physical development of preterm infants, especially for low-birth-weight infants. However, significant differences were observed at days 7 and 30, but no significant difference was observed between the two groups at day 120. The correlation analysis found similar results. SA levels at the beginning of birth were negatively correlated with infant body length and weight, but no significant correlation was observed after 120 days. All results suggested that high levels of SA in breast milk were useful for the catch-up growth of preterm infants.

Current studies on the effects of SA on infant growth and development focused on term infants and infant cognitive development. To the best of our knowledge, this report was the first large cohort study characterizing the change and influencing factors of SA concentration for a population of China. We analyzed the content and variation in SA in the breast milk of preterm and term Chinese infants at different stages and preliminarily established a database of SA in the breast milk of the Chinese population, which provided important data support for the development of formula milk powder containing SA and close to the breast milk of preterm infants.

However, there were limitations existed in this study. The 12-month postpartum SA content in the breast milk of term infants was not measured due to the small sample size at 12 months in the term infant group. In addition, because of the limited number of preterm infants, and the fact that breastfeeding combined with formula is the norm for preterm infants for 2–3 weeks postpartum due to hospitalization and other reasons, so it would be difficult to recruit if exclusive breastfeeding was required. In this study, we required preterm infants to be breastfed more than 50% of the time at 2–3 weeks postpartum, followed by exclusive breastfeeding.

In conclusion, our longitudinal study provided comprehensive data on SA from breastfeeding Chinese mothers. Similar to the previous studies, we found that with prolonged lactation time, the content of SA in breast milk gradually decreased, and the content of SA in the breast milk of preterm infants was higher than that of term infants. In addition, the SA content was associated with maternal age, pre-pregnancy BMI, and delivery mode. Moreover, the effect of SA content on infant physical development was concentrated 1 month after delivery, suggesting the importance of starting breastfeeding as early as possible after delivery.

## Data Availability Statement

The raw data supporting the conclusions of this article will be made available by the authors, without undue reservation.

## Ethics Statement

The studies involving human participants were reviewed and approved by Ethics Committee of Peking University Third Hospital. Written informed consent to participate in this study was provided by the participants' legal guardian/next of kin.

## Author Contributions

ZC and DM: conceptualization. ZC and XT: methodology. XY: software. ZC and YD: validation. ZC and LC: formal analysis. LC: investigation. YY and YX: resources. YD and YL: data curation. ZC and HL: writing—original draft preparation. YC and DM: writing—review and editing. ZC and YC: visualization. DM: supervision. YX and DM: project administration and funding acquisition. All authors have read and agreed to the published version of the manuscript.

## Funding

This research was supported by a Grant from the Natural Science Foundation of Beijing Municipality (Nos. S160004 and S170003) to XT and DM and the Peking University Third Hospital Research Fund for Outstanding Overseas Returnees (BYSYLXHG2019005) to YX.

## Conflict of Interest

YL and LC were employed by Beijing Sanyuan Foods Co., Ltd. The remaining authors declare that the research was conducted in the absence of any commercial or financial relationships that could be construed as a potential conflict of interest.

## Publisher's Note

All claims expressed in this article are solely those of the authors and do not necessarily represent those of their affiliated organizations, or those of the publisher, the editors and the reviewers. Any product that may be evaluated in this article, or claim that may be made by its manufacturer, is not guaranteed or endorsed by the publisher.
